# Dysregulation of hepatic microRNA expression in C57BL/6 mice affected by excretory-secretory products of *Fasciola gigantica*

**DOI:** 10.1371/journal.pntd.0008951

**Published:** 2020-12-17

**Authors:** Wei Shi, Jun-Jun He, Xue-Fang Mei, Ke-Jing Lu, Zi-Xuan Zeng, Yao-Yao Zhang, Zhao-An Sheng, Hany M. Elsheikha, Wei-Yi Huang, Xing-Quan Zhu

**Affiliations:** 1 State Key Laboratory of Veterinary Etiological Biology, Key Laboratory of Veterinary Parasitology of Gansu Province, Lanzhou Veterinary Research Institute, Chinese Academy of Agricultural Sciences, Lanzhou, Gansu Province, People’s Republic of China; 2 School of Animal Science and Technology, Guangxi University, Nanning, Guangxi Zhuang Autonomous Region, People’s Republic of China; 3 School of Preclinical Medicine, Guangxi Medical University, Nanning, Guangxi Zhuang Autonomous Region, People’s Republic of China; 4 Faculty of Medicine and Health Sciences, School of Veterinary Medicine and Science, University of Nottingham, Loughborough, United Kingdom; 5 College of Veterinary Medicine, Shanxi Agricultural University, Taigu, Shanxi Province, People’s Republic of China; Khon Kaen University Faculty of Medicine, THAILAND

## Abstract

The excretory-secretory products released by the liver fluke *Fasciola gigantica* (FgESPs) play important roles in regulating the host immune response during the infection. Identification of hepatic miRNAs altered by FgESPs may improve our understanding of the pathogenesis of *F*. *gigantica* infection. In this study, we investigated the alterations in the hepatic microRNAs (miRNAs) in mice treated with FgESPs using high-throughput small RNA (sRNA) sequencing and bioinformatics analysis. The expression of seven miRNAs was confirmed by quantitative stem-loop reverse transcription quantitative PCR (qRT-PCR). A total of 1,313 miRNAs were identified in the liver of mice, and the differentially expressed (DE) miRNAs varied across the time lapsed post exposure to FgESPs. We identified 67, 154 and 53 dysregulated miRNAs at 1, 4 and 12 weeks post-exposure, respectively. 5 miRNAs (miR-126a-3p, miR-150-5p, miR-155-5p, miR-181a-5p and miR-362-3p) were commonly dysregulated at the three time points. We also found that most of the DE miRNAs were induced by FgESPs in the mouse liver after 4 weeks of exposure. These were subjected to Gene Ontology (GO) enrichment analysis, which showed that the predicted targets of the hepatic DE miRNAs of mice 4 weeks of FgESPs injection were enriched in GO terms, including cell membrane, ion binding, cellular communication, organelle and DNA damage. KEGG analysis indicated that the predicted targets of the most downregulated miRNAs were involved in 15 neural activity-related pathways, 6 digestion-related pathways, 20 immune response-related pathways and 17 cancer-related pathways. These data provide new insights into how FgESPs can dysregulate hepatic miRNAs, which play important roles in modulating several aspects of *F*. *gigantica* pathogenesis.

## Author summary

Fasciolosis is a worldwide neglected parasitic disease which is caused by the infections of liver flukes, such as *Fasciola hepatica* and *Fasciola gigantica*. The excretory-secretory products released by *F*. *gigantica* (FgESPs) play important roles in regulating host biological processes. However, the regulation mechanism remains unclear. This study used high-throughput RNA sequencing to describe the comprehensive small RNA profile altered by FgESPs stimulation. We manifested that weeks of FgESPs exposure lead to a possible hypo-reactiveness and the small RNA profile was significantly altered, especially at 4 weeks post exposure. GO and KEGG pathway analyses of DEmiRNAs targets showed that FgESPs participate in regulation of neural activities, digestive function, and immune responses of host, alter several host cellular components or functions such as cell membrane, cellular communication, organelle and checking of DNA damage. The findings of this study reveal the small RNA profile changes following FgESPs stimulation, providing novel data for elucidating the interaction between *Fasciola gigantica* and infected host.

## Introduction

Fasciolosis is a worldwide parasitic disease caused by the liver flukes *Fasciola hepatica* and *Fasciola gigantica* [1]. *F*. *gigantica* is prevalent in developing countries of Asia and Africa, causing livestock production losses that exacerbates the poverty of people in these regions. Fasciolosis significantly threatens agricultural economy and public health [2]. Persistent and chronic fascioliasis in both animals and humans can be associated with disorders of the hepatobiliary system, including cholelithiasis, cholangitis, pancreatitis, cholecystitis and hepatic fibrosis [3]. The pathogenic effects of these liver flukes are caused by direct physical interaction with the host tissue and through exposure to their excretory-secretory products (ESPs). The parasite ESPs is a natural bio-active mixture of secretory proteins and extracellular vesicles secreted through the parasite’s tegument or gut and released from oral openings, including proteases, enzyme regulators, antioxidative proteins, transporters, ligand-binding proteins, retinol- and fatty acid-binding proteins, globins, etc; and still, some other molecules which remain unidentified [4–6]. Besides, the composition of the ESPs and the content of each ESP component are parasitic lifecycle stage-dependently variational [7,8]. Proteomic analysis of ESPs released by *F*. *gigantica* (hereinafter referred to as “FgESPs”) has identified over one hundred proteins, and the composition of FgESPs varies with the development of the flukes. Among which, the annotated proteins belong to cathepsin family, glutathione S-transferases, calcium-binding proteins, and some catalytic activity and cellular process-relevant proteins, and many of them correlate with the regulation of host immune response; while the functions of most of other protein components are unknown [8]. FgESPs can modulate the immune response by influencing the expression profiles of cytokines and transcriptional factors of the affected hosts [9]. However, fully understanding the composition and functions of FgESPs yet has a long way to go. Given the complexity and uncertainty of components and biological/immunological functions involved in ESPs, more researches are still required to elucidate the precise mechanisms by which FgESPs modulate the host immune response.

Numerous of studies have shown key roles of micro ribonucleic acid (miRNAs) in various biological and physiological processes in almost all eukaryotes [10–13]. The mutation and dysregulation of miRNAs can influence the transcription of the related target genes [14], and may cause diseases and even cancers in humans and animals [15–17]. MiRNA regulation has been also shown to play a role during parasite infections [18–21]. Infections with protozoan and helminthic parasites (such as *Toxoplasma gondii*, *Plasmodium chabaudi*, *Clonorchis sinensis*, *Opisthorchis viverrini*, and *Schistosoma japonicum*) [22–26], as well as the products released by the parasites (such as ESPs of *C*. *sinensis*) have been reported to alter the miRNA profiles in the affected hosts [27]. Many studies have demonstrated the roles of miRNAs in liver homeostasis and disease associated with dysregulations of miRNAs [28–31]. Exploring alteration of the host miRNA regulation can therefore provide valuable information for better understanding the pathogenic or immunological mechanisms associated with the infection. However, the connection between hepatic miRNAs regulation and *F*. *gigantica* infection remains largely unknown.

Considering the impact of *F*. *gigantica* infection on the host liver dysfunction, identification of differentially expressed (DE) miRNAs in the affected liver would provide information for revealing whether and how *F*. *gigantica* flukes use their ESPs to influence the host liver functions. Here we describe the dynamic changes in miRNA expression in the mouse liver exposed to FgESPs for 1,2 and 4 weeks, using a high-throughput sequencing and bioinformatics analyses. Our results highlight the multifaceted role of FgESPs in altering the expression of many miRNA and in *F*. *gigantica* pathogenesis.

## Methods

### Ethics statement

The study design was reviewed and approved by the Animal Ethics Committee of Lanzhou Veterinary Research Institute (LVRI), Chinese Academy of Agricultural Sciences (CAAS). The procedures involving animals were carried out in accordance with the Animal Ethics Procedures and Guidelines of the People's Republic of China.

### The parasite materials

Adult flukes of *F*. *gigantica* were collected from the bile ducts and gall bladders of buffaloes slaughtered for meat at slaughterhouses in Nanning, Guangxi, an endemic area of *F*. *gigantica* infection in China. PCR amplification and sequencing of the second internal transcribed spacer (ITS2) of ribosomal DNA was used to confirm the identity of the collected flukes as *F*. *gigantica*, as previously described [32]. The FgESPs were prepared as described previously [33,34] with slight modification. Briefly, adult worms were washed and cultured in PBSG buffer (PBS containing glucose, 1 worm/2 mL) at 37°C for 2 h. The supernatant was collected, centrifuged at 10,000× g for 30 min at 4°C and filtered through a 0.22 μm Millipore filter. The collection was then processed by vacuum freeze-drying. All procedures above strictly followed the standards of aseptic operation and used endotoxin-free reagents to avoid introducing any possible contamination or exogenous endotoxin. The endotoxin level was quantified using the ToxinSensor Chromogenic LAL Endotoxin Assay Kit (L00350, GenScript, Nanjing, China) based on at least three repeated measurements. The FgESPs was then tested major characteristic bands by SDS-PAGE, aliquoted, freeze-dehydrated, and stored at—80°C until use.

### Treatment of mice with FgESPs

Female C57BL/6 mice (six- to seven-week old) of specific pathogen free (SPF) grade, were purchased from the Laboratory Animal Center of Guangxi Medical University and housed in an air-conditioned room at 25°C under a 12-h dark/light cycle with ample standard laboratory food and water. At each time point, three mice were individually inoculated with 200 μg of FgESPs diluted with 0.5 mL of sterile phosphate buffer solution (PBS) through intraperitoneal injection at 3.5 days interval (to maintain a certain level of challenge); another three mice were injected i.p. with equal volume of sterile PBS only and served as control. The general health of the mice were monitored during the entire experiment.

### Total RNA isolation

The study was designed to examine the mouse liver after a short-term (1 week or 4 weeks) or a long-term (12 weeks) of exposure to FgESPs, in order to observe the dynamic effects of FgESPs on hepatic miRNA expression profiles. The mice were sacrificed after 1 week, 4 weeks or 12 weeks post-exposure (wpe) of FgESPs. The livers were harvested, liquid nitrogen frozen and preserved at—80°C, until RNA extraction. Total RNAs of the left lobes of liver tissues were extracted by TRIzol Reagent (Invitrogen, Carlsbad, CA, US), according to the manufacturer’s protocol. The degradation and contamination of the isolated RNA were monitored on 1% agarose gels, and the RNA purity was examined using the NanoPhotometer spectrophotometer (IMPLEN, CA, USA). Total RNA was quantified using the Qubit RNA Assay Kit in Qubit 2.0 Flurometer (Life Technologies, CA, USA), and the RNA integrity was assessed using Agilent 2100 bioanalyzer system (Agilent Technologies, CA, USA).

### Library preparation and small RNA sequencing

Three μg total RNA per sample was used as the input material for construction of small RNA library. The libraries for all groups were generated using NEBNext Multiplex Small RNA Library Prep Set for Illumina (NEB, USA). Briefly, NEB 3’ SR Adaptor was ligated to 3’-terminal of any small RNA. After the 3’-ligation reaction completed, the unreacted free 3’ SR Adaptor was hybridized by adding the SR RT Primer, and the single-strand adaptors transformed into a double-strand DNA (the step is very important to avoiding any adverse formation of adaptor-dimer). Similarly, 5’ SR Adaptor was ligated to 5’-terminals of small RNAs. For the reverse transcription, the first strand cDNA was synthesized as the PCR template by using M-MuLV Reverse Transcriptase (RNase H^-^). LongAmp Taq 2× Master Mix, SR Primer for illumina and the index primer were used for PCR amplification. PCR products were purified on a 8% polyacrylamide gel (100V, 80 min). The 140–160 bp length DNA fragments (~ 20–40 bp of sRNA plus a total 120 bp of adaptors) were selectively recovered and dissolved in 8 μL of elution buffer. The quality of sRNA library was assessed on the Agilent Bioanalyzer 2100 System (Agilent Technologies, CA, USA). The clustering of the index-coded samples was performed on a cBot Cluster Generation System using TruSeq SR Cluster Kit v3-cBot-HS (Illumia). The sRNA libraries were then sequenced on an Illumina Hiseq 2500/2000 platform. 50 bp single-end reads were generated for further analyses.

### Data processing and miRNA annotation

Raw reads from sRNA sequencing were filtered out with NGQC software (Novogene) if they met any of the exclusion criteria (N% > 10, quality score < 50%, missing insert, 5′ adaptor contamination, 3′ adaptor absence, contained poly-A, G, C, or T). Clean reads of 15~ 35 bp in length were used for further analysis. The mouse reference genome (ftp://ftp.ensembl.org/pub/release-89/fasta/mus_musculus/dna) was used for sequence alignment. The reads originating from protein-coding genes, repeat sequences, rRNA, tRNA, snRNA, and snoRNA were screened by mapping with entries in the Rfam database (http://rfam.xfam.org). The remaining sequences were considered as candidate miRNAs.

To search the known miRNAs, we used a modified version of mirdeep2 [34] (https://www.mdc-berlin.de/8551903/en/) and srna-tools-cli (http://srna-tools.cmp.uea.ac.uk/) based on miRBase 20.0 as a reference (http://www.mirbase.org), to obtain the potential miRNA and predict the secondary structures. To predict novel miRNA, miREvo [35] (http://evolution.sysu.edu.cn/software/mirevo.htm) and mirdeep2 [36] were used through exploring the characteristics of the secondary structures, the Dicer cleavage sites, and the minimum free energy of the sRNA unannotated in the former steps. The recommended default parameters were applied for this analysis.

### Differential expression analysis

Differential expression miRNA between different treatment periods was identified using the DESeq R package (1.8.3). *P*-values of 0.05 (default value) was set as the significance threshold for differential expression. Quantification of miRNA expression levels were estimated by transcript per million tags (TPM) using the normalization formula [37]: Normalized expression = mapped readcount/Total reads*1000000. For multivariate analysis, data was imported into SIMCA software (version 13.0.3, Umetrics, Umeå, Sweden), and a multivariate partial least squares discriminant analysis (PLS-DA) was performed to evaluate whether the miRNA profile gained in different time groups had similar performance induced by variable duration of FgESPs exposure.

### MiRNA targeted gene prediction and bioinformatics analysis

To predict the target genes of DEmiRNAs, the online available software miRanda [38] (http://sanderlab.org/tools/micrornas.html/), PITA [39] (http://genie.weizmann.ac.il/pubs/mir07/mir07_data.html) and RNAhybrid [40] (http://bibiserv.techfak.uni-bielefeld.de/rnahybrid) were used to identify potential miRNA binding sites. Gene Ontology (GO) (http://www.geneontology.org) was used for gene functional enrichment of predicted target gene of DEmiRNA. GOseq based Wallenius non-central hyper-geometric distribution was applied for GO enrichment analysis. Kyoto Encyclopedia of Genes and Genomes (KEGG) data (http://www.kegg.jp/kegg/) was used for analyzing the roles (including physiological functions and predicted signaling pathway) of target genes. With the predicted target genes, we performed KEGG pathways analysis using KOBAS software to determine the statistical enrichment of certain pathways [41].

### Validation of sequencing data by qRT-PCR analysis

DEmiRNAs were validated using quantitative stem-loop reverse transcription quantitative PCR (qRT-PCR) with SYBR green and specifically designed primers (**Table 1**). U6 RNA served as a housekeeping reference miRNA for normalization of targeted miRNA expression [42]. The RNA templates for qRT-PCR analysis were the same total RNA samples used for RNA-seq. Total RNA from tissues was reverse-transcribed to cDNA using RT primers and the ImProm-II Reverse Transcription System (Promega, Madison, WI, USA). Each 20-μL qPCR reaction contained 10 μL of SYBR GREEN qPCR Super Mix (Invitrogen, Carlsbad, CA, USA), 0.8 μL of a forward/reverse primer mixture, 1 μL of cDNA template, and 8.2 μL of RNase free water. The cycling protocol of in two-step qRT-PCR was as follows: 95°C for 2 min for activating the hot-start enzyme, followed by 40 cycles of 95°C for 15 s and 60°C for 30 s, and then by dissociation run from 60°C to 95°C for melting curve analysis. All assays were set with triplicate and miRNA relative quantification was calculated using the 2^–ΔΔCq^ method [42].

**Table 1:** Primer sequences for miRNA reverse transcription quantitative PCR.

Primer name	Sequence (5' to 3')
mmu-let-7a-5p-F	ACACTCCAGCTGGGTGAGGTAGTAGGTTGT
mmu-miR-10a-5p-F	ACACTCCAGCTGGGTACCCTGTAGATCCGAAT
mmu-miR-122-5p -F	ACACTCCAGCTGGGTGGAGTGTGACAATGGTG
mmu-miR-143-3p-F	ACACTCCAGCTGGGTGAGATGAAGCACTGTAG
mmu-miR-150-5p-F	ACACTCCAGCTGGGTCTCCCAACCCTTGTACC
mmu-miR-155-5p-F	ACACTCCAGCTGGGTTAATGCTAATTGTGAT
mmu-miR-29a-3p-F	ACACTCCAGCTGGGTAGCACCATCTGAAATC
*miRNA-R	CTCAACTGGTGTCGTGGA
U6-F	CTCGCTTCGGCAGCACA
U6-R	AACGCTTCACGAATTTGCGT

*miRNA-R serves as the universal reverse primer for all miRNAs in the present study.

## Results

### Overview features of small RNA sequencing data

**Fig 1:**
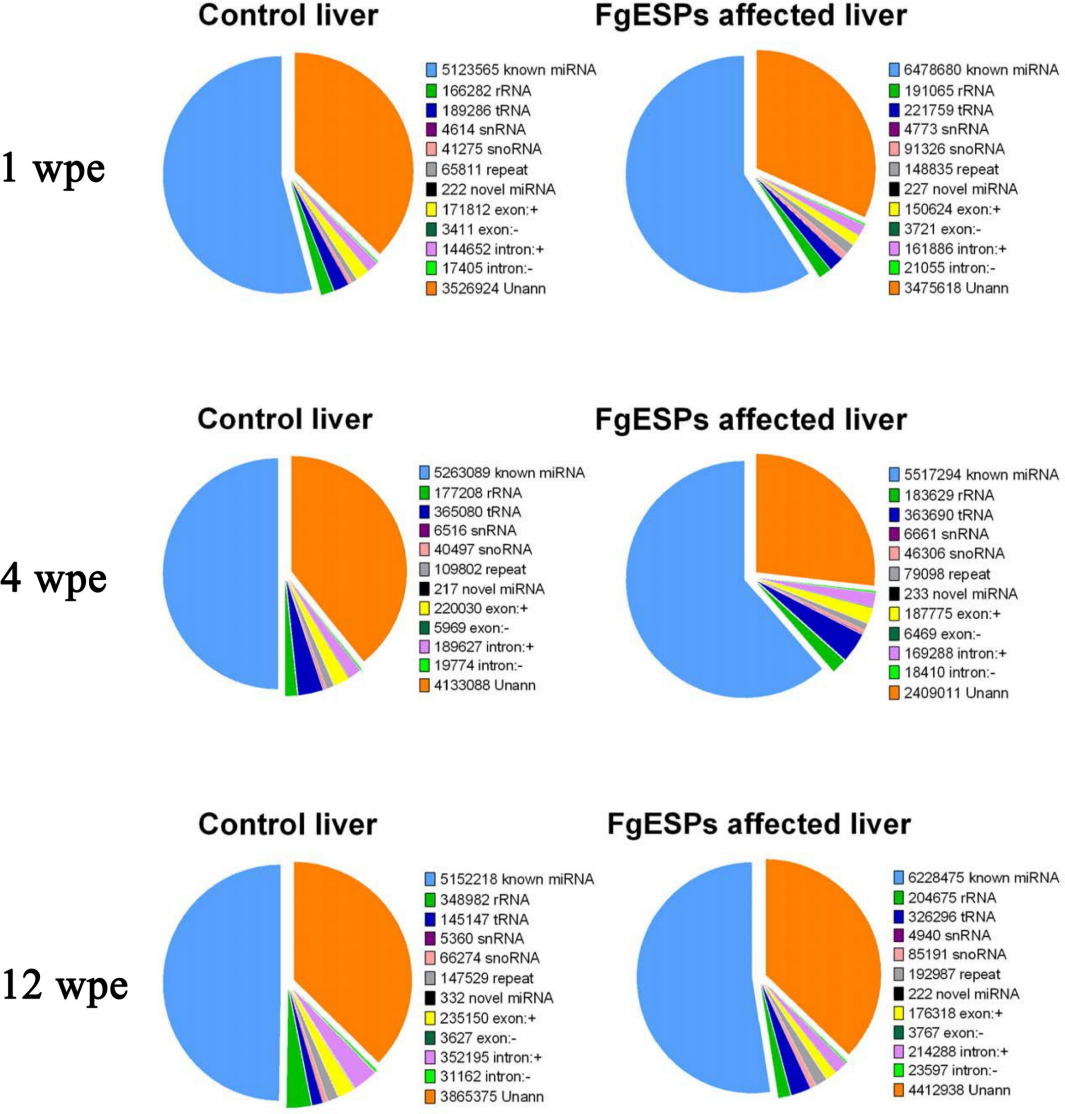
Frequency of unique sRNA distribution among the different categories. The unique sequences were subjected to searches for the types and numbers of sRNA using the Rfam databases (rRNA, tRNA, snRNA, snoRNA, known miRNAs, novel miRNAs, and other noncoding RNA, etc.).

In this study, 200 μg of FgESPs preparation with 48.15 EU/mL of endotoxin was used to continuously inject the mice for three different periods. No visible alteration of physical and mental sign was observed in all mice treated or untreated with FgESPs for 1, 4 or 12 weeks, suggesting no or very mild biological toxicity of FgESPs treatment to mice general health condition in this study. Mice liver tissues from groups of three different test time periods (1, 4 and 12 weeks) were sequenced simultaneously by Illumina system. The sequencing error rate of each one single base was calculated according to the Phred quanlity score (Q-score) for prediction of base-calling error probability [42,43]. As for quality control, Q20, Q30 and GC-content of the raw data were calculated. In our study, more than 97% reads up to Q20 standard, and 94% reads up to Q30 standard; and the GC-content was between 48.52%-49.97% (shown in **S1 Table**), indicating our sequencing data of high quality. After filtering out the low-quality reads and meaningless reads, each individual sample acquired in average over 11 million clean reads (ranging from 10,069,052 to 15,127,517). By mapping with the reference mouse genome (GRCm38.p5), over 87% of sequenced clean reads from each sample were perfectly mapped to the mouse genome (**S2 Table**). In addition, the types and number of sRNAs were identified using Rfam databases (rRNA, tRNA, snRNA, snoRNA, known miRNAs, novel miRNAs, and other noncoding RNA). For all tested samples, an average of 55.0% of the unique sequences were defined as miRNAs, less than 0.01% were novel miRNAs, 35% were unannotated, and the rests were other known categories of identified small RNA, including rRNA, tRNA, snRNA, snoRNA, gene intron/exon, etc (**Fig 1**). It showed that mapped known miRNA accounted for very large proportion in the sequenced reads. A total of 1418 miRNAs (1313 known mature miRNAs and 105 novel miRNAs) were detected in all test samples.

### Differentially expressed hepatic miRNAs

**Fig 2:**
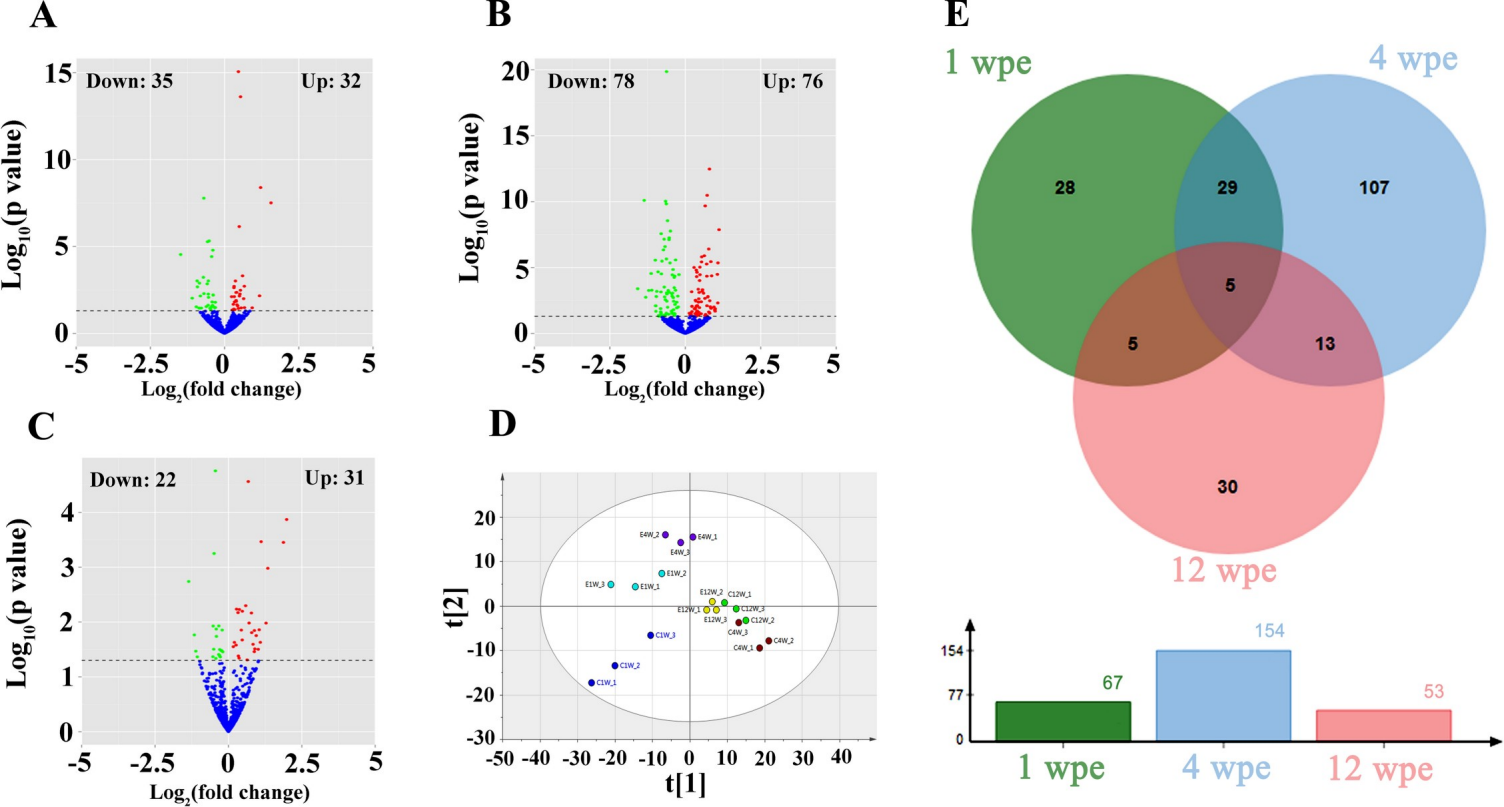
Summary of the distribution of DEmiRNAs. (A-C) Volcano plots of DEmiRNAs at 1, 4 and 12 wpe, respectively. (D) PLS-DA plot of miRNA profiles at 1, 4 and 12 wpe. (E) Venn plot of DEmiRNAs at 1, 4 and 12 wpe. Log2 (Fold change) = Log2(Relative expression of treated liver/ Relative expression of non-treated liver).

A total of 67, 154 and 53 miRNAs were differentially expressed in mice livers at 1, 4 and 12 wpe, respectively (**Figs 2A, 2B, 2C** and **S1**). Details of DEmiRNAs are listed in **S3 Table**. Among these, 32 upregulated- and 35 downregulated- hepatic miRNAs showed statistically significant after one week’s exposure, while 76 upregulated miRNAs and 78 downregulated miRNAs were identified at 4 wpe. After a longer period of FgESPs’ exposure (12 weeks), there were 31 miRNAs upregulated and 22 miRNAs downregulated. Among the known miRNAs, miR-708-3p was upregulated most by 1.9 (Log2 fold-change), while miR-215-5p was downregulated most by -1.4 (Log2 fold-change). As shown in **Fig 2D**, PLS-DA modeling on miRNAs from three groups demonstrated that loading dots representing individual FgESPs-treated or untreated samples in 1 wpe group and 4 wpe group, were separated in different quadrants of the diagram, which suggested that the FgESPs induced distinct miRNA profiles during the early stage of immunization. In contrast, loading dots of treated samples and control samples from 12 wpe group were distributed in the close area and mapped to boundary between first quartile and third quartile. The Venn diagram and column chart in **Fig 2E** summarized the number of DEmiRNAs in the mouse livers from above three groups, demonstrating the most DEmiRNAs were gained in 4 wpe rather than the other two time points. Five of DEmiRNAs were found in all three groups, including miR-126a-3p, miR-150-5p, miR-155-5p, miR-181a-5p and miR-362-3p, although they presented different variation trends.

### Target gene prediction and GO annotation analyses

**Fig 3:**
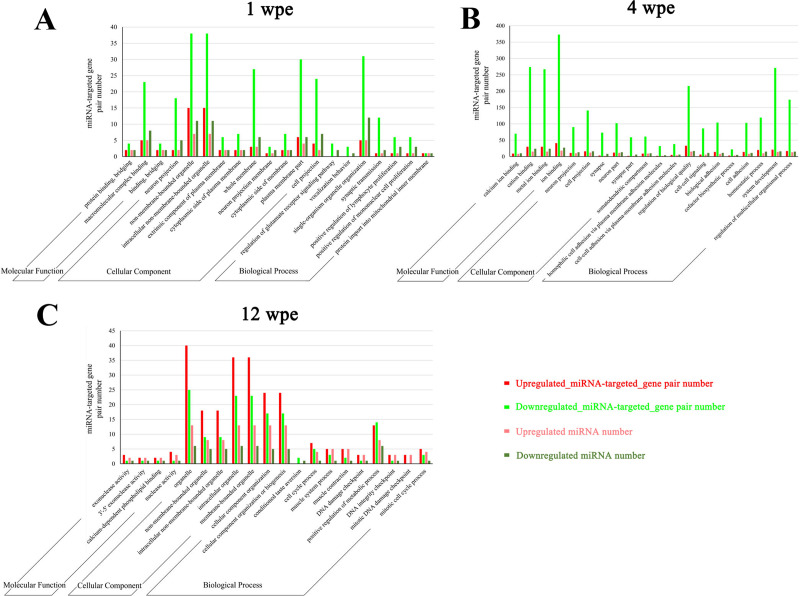
GO enrichment analysis of the targets of DEmiRNAs. According to *p* value, the top 20 GO terms belonging to the categories biological process, molecular function and cellular component are shown.

According to FDR corrected *p* value of the enrichment analysis, top 20 enriched GO annotations of predicted target genes of DEmiRNAs from all three time groups are shown in **Fig 3**. This information revealed that some potentially important biological functions in the host liver reacted with the injected FgESPs. Most enriched GO terms of 1 wpe were mainly involved in cell membrane or organelle (e.g., whole membrane, non-membrane bounded organelle, neuron projection membrane) **(Fig 3A)**. Most enriched GO terms of 4 wpe were mainly involved cellular connection, ion binding, cation binding, cell projection, synapse and cell-cell signaling **(Fig 3B)**. In contrast, most enriched GO terms of 12 wpe were mainly involved in organelles, cellular component organization or biogenesis, DNA integrity checkpoint, DNA damage checkpoint and cell cycle process (**Fig 3C**). Additionally, most enriched GO terms were targeted by downregulated miRNAs in GO analysis for samples from 1 wpe and 4 wpe groups, while GO term categories were enriched in upregulated miRNAs by 12 wpe.

### KEGG pathway analysis

**Fig 4:**
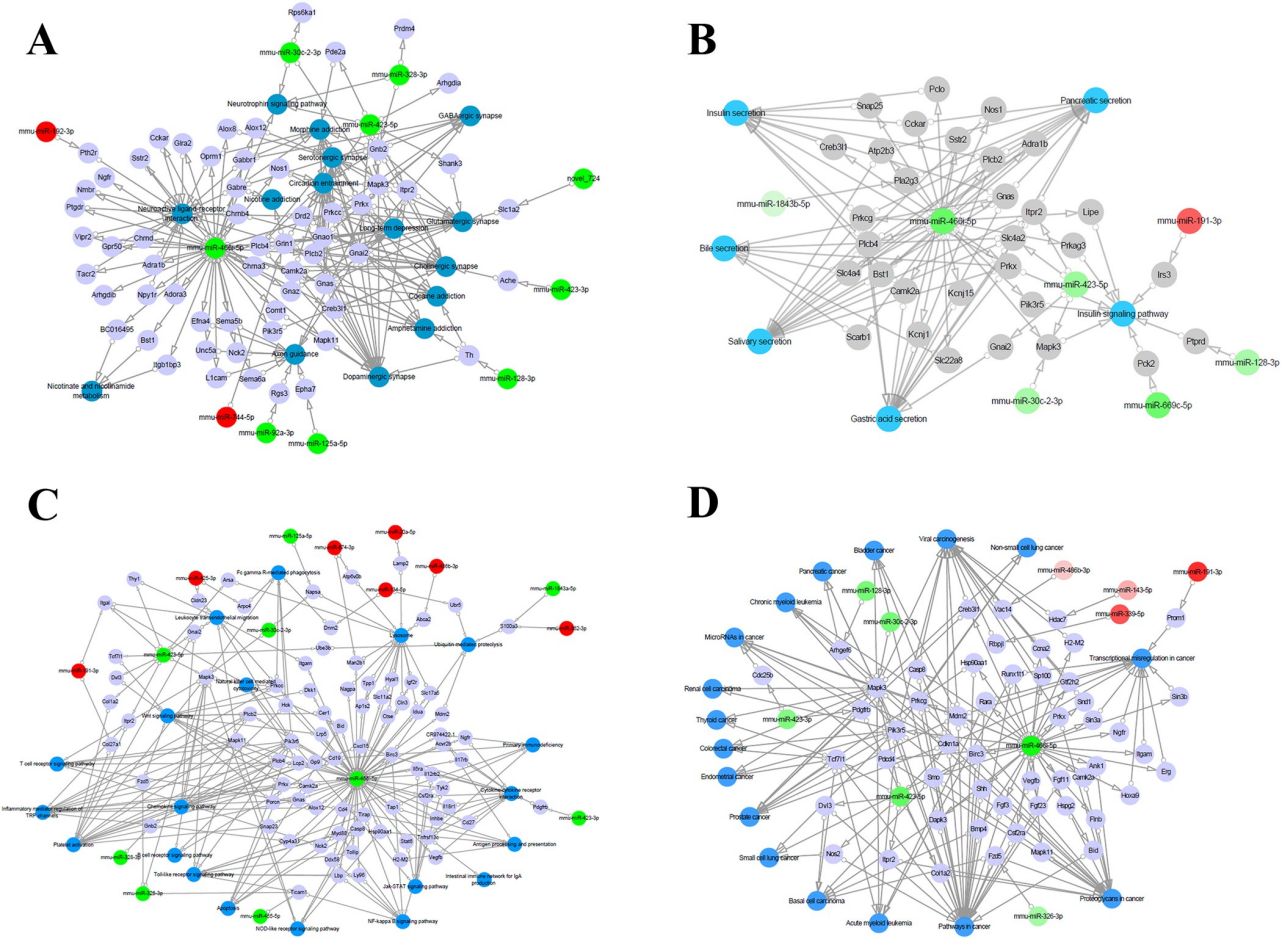
Interaction between DEmiRNAs and their target genes in the pathways related to (A) neural activities, (B) food digestion, (C) immune responses and (D) cancer. Blue dots represent the pathways, gray dots represent the targets of DEmiRNAs, green dots represent the downregulated miRNAs, and red dots represent the upregulated miRNAs.

KEGG analyses indicated that 15 pathways related to neural activity, 6 pathways related to food digestion, 20 pathways related to immune response, and 17 pathways related to cancer, were subject to regulation by the DEmiRNAs (**Table 2**). Note that the 4 wpe group has the most DEmiRNA or DEmiRNA targets that mapped to neuro, digesting, immune and cancer pathways (**Table 2** and **Fig 4**). Among these pathways of 4 wpe, pathways in cancer, viral carcinogenesis, glutamatergic synapse, cholinergic synapse, axon guidance, insulin signaling, lysosome, Toll-like receptor signaling pathway and Fc gamma R-mediated phagocytosis were targeted by more than 5 DEmiRNAs. The cancer-related pathways were the top pathway that mapped with 26 target genes of DEmiRNAs at 4 wpe.

**Table 2:** KEGG pathway enrichment analysis.

Pathways	DEmiRNA-targeted gene pair number						DEmiRNA number					
	1 wpe		4 wpe		12 wpe		1 wpe		4 wpe		12 wpe	
	Up	Down	Up	Down	Up	Down	Up	Down	Up	Down	Up	Down
**Neural activity-related pathways**												
Glutamatergic synapse	1	6	1	13	0	1	1	3	1	5	0	1
Morphine addiction	1	5	0	10	0	0	1	3	0	3	0	0
Cocaine addiction	0	3	1	6	0	1	0	1	1	3	0	1
Cholinergic synapse	1	2	0	16	1	2	1	2	0	5	1	2
Axon guidance	1	3	0	11	1	1	1	2	0	5	1	1
Long-term depression	0	2	0	11	0	0	0	1	0	3	0	0
Serotonergic synapse	1	3	0	13	0	0	1	2	0	4	0	0
Neuroactive ligand-receptor interaction	0	5	2	18	0	1	0	2	2	2	0	1
GABAergic synapse	1	3	0	7	0	0	1	3	0	3	0	0
Circadian entrainment	1	3	1	12	0	2	1	2	1	4	0	1
Dopaminergic synapse	1	4	0	15	1	3	1	3	0	4	1	2
Nicotine addiction	0	1	1	1	0	1	0	1	1	1	0	1
Amphetamine addiction	0	1	1	6	0	2	0	1	1	2	0	1
Nicotinate and nicotinamide metabolism	0	0	0	3	0	0	0	0	0	1	0	0
Neurotrophin signaling pathway	0	0	0	10	1	2	0	0	0	4	1	2
**Digestion-related pathways**												
Bile secretion	0	1	0	6	0	0	0	1	0	2	0	0
Insulin secretion	0	2	0	10	0	1	0	1	0	1	0	1
Insulin signaling pathway	0	1	1	9	1	1	0	1	1	5	1	1
Gastric acid secretion	0	2	0	12	0	1	0	1	0	2	0	1
Salivary secretion	0	3	0	11	0	0	0	1	0	2	0	0
Pancreatic secretion	0	3	0	11	0	0	0	1	0	3	0	0
**Immune response-related pathways**												
Lysosome	1	3	4	14	0	0	1	2	3	4	0	0
Platelet activation	0	2	1	14	1	1	0	1	1	3	1	1
Cytokine-cytokine receptor interaction	0	1	0	13	0	0	0	1	0	2	0	0
Toll-like receptor signaling pathway	0	3	0	12	1	1	0	3	0	5	1	1
Wnt signaling pathway	0	2	0	12	0	1	0	2	0	3	0	1
Inflammatory mediator regulation of TRP channels	0	2	0	11	0	1	0	1	0	2	0	1
Chemokine signaling pathway	1	2	0	10	1	1	1	2	0	4	1	1
Leukocyte transendothelial migration	0	2	2	7	0	1	0	1	2	2	0	1
NF-kappa B signaling pathway	0	1	0	9	0	0	0	1	0	3	0	0
Natural killer cell mediated cytotoxicity	0	0	1	7	0	1	0	0	1	3	0	1
Fc gamma R-mediated phagocytosis	0	1	2	5	1	1	0	1	2	3	1	1
T cell receptor signaling pathway	0	0	0	7	1	1	0	0	0	3	1	1
NOD-like receptor signaling pathway	0	1	0	6	0	1	0	1	0	3	0	1
Apoptosis	0	1	0	6	1	1	0	1	0	1	1	1
Ubiquitin mediated proteolysis	1	0	2	4	1	0	1	0	2	3	1	0
Jak-STAT signaling pathway	0	0	0	6	1	1	0	0	0	1	1	1
Primary immunodeficiency	0	2	0	5	0	0	0	2	0	1	0	0
Antigen processing and presentation	0	1	0	5	0	0	0	1	0	1	0	0
B cell receptor signaling pathway	0	0	0	4	1	1	0	0	0	3	1	1
Intestinal immune network for IgA production	0	1	0	1	0	0	0	1	0	1	0	0
**Cancer-related pathways**												
Basal cell carcinoma	0	2	0	6	0	0	0	2	0	3	0	0
Transcriptional misregulation in cancer	0	2	1	11	0	1	0	2	1	2	0	1
Proteoglycans in cancer	1	2	0	17	2	2	1	2	0	4	2	2
Viral carcinogenesis	0	2	3	13	0	0	0	2	3	3	0	0
Pathways in cancer	0	3	0	26	1	1	0	3	0	5	1	1
Bladder cancer	0	0	0	5	0	0	0	0	0	3	0	0
Acute myeloid leukemia	0	0	0	6	1	1	0	0	0	3	1	1
Thyroid cancer	0	0	0	3	0	0	0	0	0	2	0	0
Endometrial cancer	0	0	0	4	1	1	0	0	0	3	1	1
Chronic myeloid leukemia	0	0	0	5	1	1	0	0	0	3	1	1
Non-small cell lung cancer	0	0	0	4	1	1	0	0	0	3	1	1
Pancreatic cancer	0	0	0	4	1	1	0	0	0	4	1	1
Small cell lung cancer	0	0	0	3	1	1	0	0	0	2	1	1
Renal cell carcinoma	0	0	0	3	1	1	0	0	0	3	1	1
MicroRNAs in cancer	1	0	0	6	0	0	1	0	0	3	0	0
Colorectal cancer	0	0	0	4	1	1	0	0	0	3	1	1
Prostate cancer	0	0	0	9	1	1	0	0	0	4	1	1

### Validation of selected miRNAs by qRT-PCR

**Fig 5:**
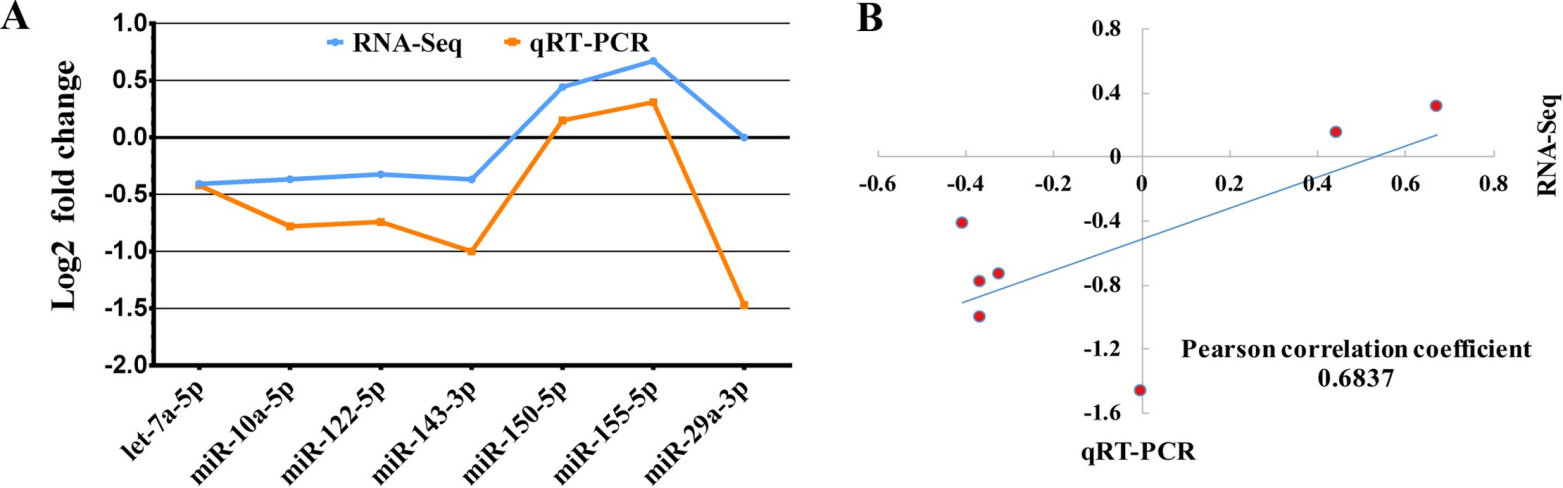
qRT-PCR validation of seven DEmiRNAs obtained by small RNA sequencing analysis of mouse livers 4-week post treatment with FgESPs. (A) Bar plot of validation result. (B) Pearson correlation analysis between qRT-PCR and RNA-seq. Log2(Fold change) = Log2(Relative expression of treated liver/ Relative expression of non-treated liver).

As shown in **Fig 5**, the expression patterns of selected miRNAs (let-7a-5p, miR-10a-5p, miR-122-5p, miR-143-3p, miR-150-5p, miR-155-5p and miR-29a-3p in the 4 wpe group) were validated by qRT-PCR, and the result was mostly coincide with the patterns of our sequencing data, and the pearson correlation index between qRT-PCR and RNA-seq was 0.6837. It indicated that the miRNA sequencing results were reliable and appropriate for further analysis. Note that 4 weeks’ exposure to FgESPs induced upregulation of miR-150-5p and miR-155-5p expression in the mouse liver, but decreased expression of the other tested miRNAs.

## Discussions

*Fasciola gigantica* is prevalent in developing countries and threatens the health in both animals and humans. Parasitic ESPs have regulatory roles in the interaction between host and parasite. However, the components and functions of ESPs of *F*. *gigantica* remain to be determined. To explore the functions of FgESPs, in this study, we employed a high-throughput RNA sequencing approach to identify hepatic DEmiRNAs altered by raw/whole FgESPs over 1, 2 and 4 weeks after exposure. GO analysis clearly showed that liver responses to FgESPs may be time-dependent, affecting intercellular interactions through manipulating the host hepatic miRNAs. Unlike 1 wpe and 4 wpe, DEmiRNAs at 12 wpe were much fewer in number, and most of them were upregulated and enriched in organelles, cellular component organization or biogenesis, cell cycle process and DNA damage related GO terms. Only 5 miRNAs (including miR-126a-3p, miR-150-5p, miR-155-5p, miR-181a-5p and miR-362-3p) were commonly dysregulated among treated livers at all three time points compared to the untreated controls, revealing their important roles in the interplay between liver and FgESPs. One interesting finding is that all commonly dysregulated miRNA regulate the cellular proliferation. The down-regulation of miR-126a-3p in endothelial cells has been reported to promote apoptosis, and decrease proliferation and migration [44]; while the upregulation of miR-150-5p, miR-181a-5p, miR-155-5p and miR-362-3p was associated with cellular migration and/or cellular proliferation [45–48]. The participation of all these miRNAs in cellular proliferation or migration suggests that they may play roles in liver pathological changes induced by the liver flukes, warranting further investigations. The predicted targets, enriched pathways and the possible roles of DEmiRNAs in liver induced by exposure to FgESPs for 4-weeks are shown in **Table 2 and Fig 4**.

KEGG analysis provides important information about the possible influences of miRNA profile changes on the host system through the signaling pathway enrichment of miRNA target genes. As shown in **Fig 4A**, the predicted targets of hepatic DEmiRNAs induced by 4-week exposure to FgESPs in the mice were enriched in 15 neural activity-related pathways **(**listed in **Table 2)**. Neurological fasciolosis has been widely reported [49,50], however, the mechanisms that trigger the neurological manifestations remain unknown. It is believed that the neurogenic disease (e.g., cephalalgias) is a consequence of neurovirulent damages induced by the erratic migration of the flukes in the nervous system. Synapses play a role in the neural network [51]. Based on our analysis, four important synapses involved in the neuro pathways, namely glutamatergic synapse, cholinergic synapse, GABAergic synapse and dopaminergic synapse, were targeted by 8 DEmiRNAs, including miR-30c-2-3p, miR-128-3p, miR-328-3p, miR-423-3p, miR-423-5p, miR-466i-5p, miR-744-5p and novel_724. Among these miRNAs, most were predicted to target glutamatergic synapse and cholinergic synapse. Mapk3 is involved in pathways of cholinergic synapse and glutamatergic synapse, and closely related to the assembly, shape, function, plasticity and autism spectrum disorders of synapses [52]. Our analysis also found downregulated miR-30c-2-3p and miR-423-5p, both of which target the Mapk3, and this may mediate the dysfunction of Mapk3-related pathways in response to FgESPs. In addition, miR-466i-5p targeted the most genes involved in neural activities (**Fig 4A**), indicating its possible important role in neural regulation. The enrichment of these neural pathways indicates that FgESPs can modified host miRNAs that participate in neural pathways.

*Fasciola gigantica* causes damage to the host organs or tissues by invasion and migration across the gastrointestinal tract, liver parenchyma and biliary ducts, thus influencing the host’s food digestion ability. **Fig 4B** shows the digestion related pathways were enriched by target genes of DEmiRNAs relative to functions such as bile secretion, insulin secretion, insulin signaling pathway, gastric acid secretion, salivary secretion, and pancreatic secretion **(**listed in **Table 2)**. Liver flukes mainly settle in the intrahepatic bile ducts, and as such are expected to impair bile synthesis and secretion, as well as the biliary amino acids [53–55]. In our study, two downregulated miRNAs, miR-466i-5p and miR-423-5p, which targets 6 genes, were significantly enriched in bile secretion pathway at 4 wpe. These findings suggest that FgESPs can affect the expression of miRNA that target the genes involved in biliary secretion and liver functions. Moreover, some miRNAs, for example, miR-148a-3p (which has suppressive function on cell proliferation and is associated with liver fibrosis [56,57]) and miR-126 homologs (which is known to correlated with chronic hepatic inflammation [57]), were significantly differentially expressed in mice livers after continuous FgESPs administration of 4 weeks (**S3 Table**), suggesting the involvement of FgESPs with liver fibrosis tendency. Given that the causal link between parasitic ESPs and tissue fibrosis has been widely studied [58–60], the prolonged exposure of the liver to FgESPs may probably result in chronic liver diseases that could lead to digestive dysfunction.

Suppression of FgESPs to the host immune system has been studied [9]. However, an active pro-inflammatory immune response may occur in the host to counter the early infection of *F*. *gigantica*, where the predominance of specific antibodies, eosinophils and macrophages are involved [61]. Our previous study showed a significant increase of Th2 cytokines expression at 4 weeks after *F*. *gigantica* infection in water buffaloes, which is important in controlling excessive inflammation [62]. It is widely accepted that miRNAs are involved in the regulation of immune responses [63,64]. In our study, we detected 20 immune response-related pathways targeted by a larger number of down-regulated hepatic miRNAs at 4 wpe (**Fig 4C** and **Table 2**). Further investigation of the DEmiRNA profile especially at 4 wpe, may be a key to development of effective strategies to eliminate liver flukes from the host during early infection.

Among the enriched immune response-related pathways, those involving Toll-like receptor (TLRs) recognition, antibody production, as well as cytokine and chemokine secretion, are important. TLRs expressed on the immune cell surfaces are considered as the front-line of the host’s innate immune defense [65,66]. *F*. *gigantica* is known to alter the TLRs expression profile in order to evade the host’s immune attacks during infection in buffaloes [55,62]. However, the roles of FgESPs in the alteration of TLRs signaling pathways mediated by miRNA is unclear. In the present study, a total of 5 downregulated miRNAs (miR-30c-2-3p, miR-423-5p, miR-466i-5p, miR-455-5p and miR-326-3p) targeting 12 TLRs signaling pathway-related genes were identified in the livers at 4 wpe. Among these, miR-30c-2-3p and miR-423-5p were found to target the mitogen-activated protein kinase Mapk3 (also ERK1), which contributes to a signaling cascade that regulates cellular processes in response to extracellular signals and pathogens mediated by TLR recognitions [67,68]. Our data suggest that upregulation of TLRs signaling in liver mediated by exposure to FgESPs may facilitate a rapid recognition of liver fluke juveniles by the immune system during early infection. Genes, such as Dnm2, Arpc4, Pik3r5, Hck and Prkcc targeted by miR-134-5p, miR-425-3p and miR-466i-5p, were enriched in B cell receptor signaling pathway and Fc gamma receptor-mediated phagocytosis that is involved in antibody responses. Downregulation of these miRNAs may lead to functional enhancement of immune receptors, particularly antigen presentation, regulation of innate immune effector cell (including B cells) activation, and regulation of antibodies production [69,70].

Despite TLRs recognition and antibody production, the secretion of cytokines and chemokines by the host’s immune cells also plays an essential role in protecting the host [71,72]. Chemokines are released in response to the parasitic ESPs [73,74]. However, roles of chemokines associated with *F*. *gigantica* infection was rarely reported. In our study, dysregulated miRNAs, including miR-30c-2-3p, miR-328-3p, miR-423-3p, miR-423-5p and miR-466i-5p, were found to target the genes associated with chemotactic effects, *e*.*g*., Mapk3, Plcb4, Plcb2, Pik3r5, Prkx, Hck, CXCL15, Gnai2 and Gnb2. These miRNAs were also enriched in pathways involved in the functions of chemokine signaling, cytokine-cytokine receptor interaction, and leukocyte transendothelial migration. Downregulation of these miRNAs can promote the expression of their potential targets, inducing the recruitment of eosinophils and macrophages. Previous studies showed that FgESPs can regulate host immune response [75], however, host miRNA response against FgESPs remain unclear. In contrast with previous studies, our results revealed that short-term interaction with FgESPs may activate host immune responses via regulating miRNA expression.

Many studies have shown that miRNA alterations correlate with various human cancers [16,17]. *Fasciola* infection in humans is associated with chronic liver diseases, liver fibrosis and cirrhosis, but the correlation between these flukes and cancer is not yet conclusive [3,76]. As shown in **Fig 4D**, 12 cancer-related pathways were enriched, including 10 cancers (basal cell carcinoma, bladder cancer, acute/chronic myeloid leukemia, thyroid cancer, endometrial cancer, lung cancer, pancreatic cancer, renal cell carcinoma, colorectal cancer and prostate cancer) which are listed in **Table 2**. We also found additional 52 genes targeted by 9 DEmiRNAs (miR-143-5p, miR-191-3p, miR-326-3p, miR-339-5p, miR-423-3p, miR-423-5p, miR-466i-5p, miR-30c-2-3p and miR-486b-3p) were enriched in 5 pathways involved in carcinogenic pathways that mediate the initiation and progression of cancers, including transcriptional dysregulation in cancer pathway, proteoglycans in cancer pathway, viral carcinogenesis pathway, microRNAs in cancer pathway and pathways in cancer pathway. Although some DEmiRNAs participate in carcinogenic pathways, at present, no evidence is sufficient to conclude the correlation between FgESPs and carcinogenesis in the liver. It is possible that carcinogenic process is more complex and involves other carcinogens besides alteration of miRNAs.

Notably, the miR-466i-5p was found to target genes enriched in multiple pathways involved in digestion, neural activity, immune response and cancer, as described above. MiR-466i-5p has been reported to be mainly involved in the suppression of inflammation and chronic fibrosis in the liver [77,78]. Increased expression of miR-466i-5p has been found also associated with infection with some parasites (*e*.*g*., *Toxoplasma gondii* and *Plasmodium chabaudi*) [79,80]. Hence, the remarkable downregulation of miR-466i-5p enriched in multiple pathways in this study might potentially influence the function of genes that regulate several biological processes in the liver, and may serve as an anti-inflammatory molecules for reducing liver damage during exposure to FgESPs.

Although we have tried to avoid the external contamination during FgESPs preparation, endotoxin (lipopolysaccharide/LPS) was still detectable from the FgESPs used in the present study (the endotoxin is 48.15 EU/mL, which is much lower than endotoxin level in ESPs of a similar study by Falcón *et al*. [34] that we referenced for our ESPs preparation). Therefore, the possibility of that endotoxin derived from *F*. *gigantica* can not be excluded. Considering there might be parasite-derived endotoxin which should be counted an inherent component of ESPs, we did not conduct the endotoxin removal, as the same way of some similar studies [34,81]. The involvement of endotoxin with parasite ESPs in regulating host biological and immunological functions should not be ignored, especially during the serious parasite infection [82]. It has been found that LPS increases the expression of mature miR-122 in mice [83]. However, in our study, both the RNA sequencing and qRT-PCR results confirmed a mild decrease of miR-122-5p which showed opposite trend with that of endotoxin stimulation (**Fig 5** and **S3 Table**), suggesting that the influence of such minute amount of endotoxin within our prepared FgESPs could be trivial, or the expression of miR-122 was coordinated by both FgESPs and the endotoxin. For the limitation of this study, we can not distinguish the impacts of FgESPs and the endotoxin on mice liver. However, in this study, using the FgESPs without endotoxin removal reveals the global influence of natural FgESPs on the expression of miRNA of mice liver.

## Conclusions

The present study identified the dynamic global miRNA expression pattern in mice livers triggered by short- or long-term exposure to FgESPs, based on high-throughput sequencing approach. Bioinformatics analysis revealed that FgESPs as a complex mixture of parasitic antigens have the capability to modulate host gene expression by directly regulating the expressions of miRNA, especially at 4 wpe. GO and KEGG pathway analyses revealed that these DEmiRNAs participate in regulation of neural activities, digestive function, and immune responses. Our data showed that the function of the mouse livers can be compromised by natural FgESPs and revealed new clues about the mechanisms that mediate the interactions between *F*. *gigantica* and the host. Our study contributes to a more in-depth understanding of complex parasite-host relationships, which may definitely benefit downstream researches on the pathogenesis and therapeutic agent development to control *F*. *gigantica* infection in animals and humans.

## Supporting information

S1 Fig Heatmap showing DEmiRNA abundance in the liver samples at different times after exposure to FgESPs. Each column represents individual time group (1, 4 or 12 weeks post i.p. treatment with FgESPs) and each row represents DEmiRNA at 1, 4 or 12 weeks post i.p. treatment with FgESPs.
(PDF)

S1 Table The quality features of small RNA sequencing data. (DOCX)

S2 Table Sequence alignment to the reference mouse genome. (DOCX)

S3 Table The differentially expressed miRNAs in the liver tissues of C57BL/6 mice treated with FgESPs. (DOCX)
