# Supplementary figures and images for: Dysregulation of hepatic microRNA expression in C57BL/6 mice affected by excretory-secretory products of Fasciola gigantica
Source: PLoS Negl Trop Dis. 2020 Dec 17;14(12):e0008951. doi: 10.1371/journal.pntd.0008951 (PMC7775122; doi:10.1371/journal.pntd.0008951)

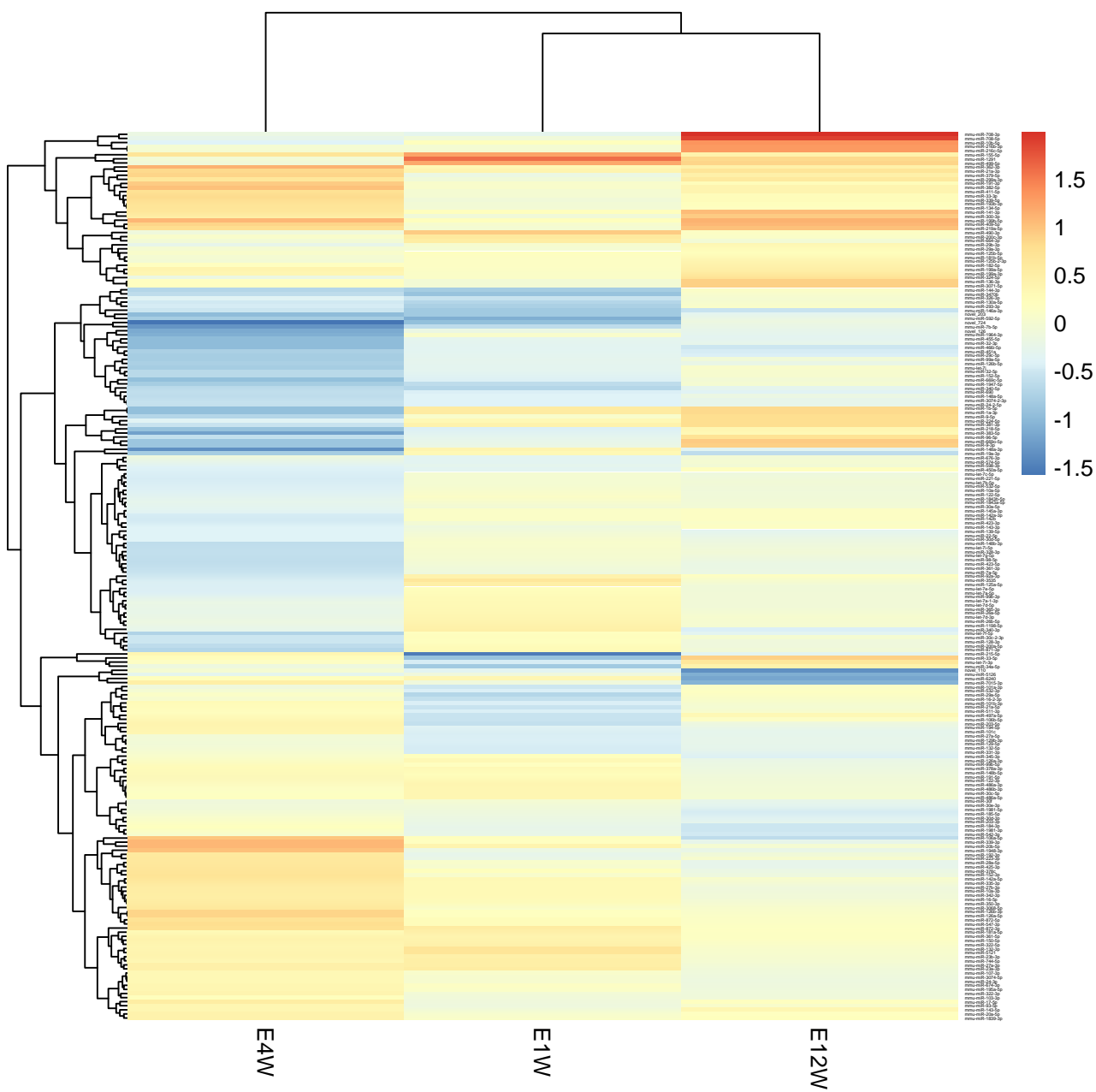

Supplement: S1 Fig — Each column represents individual time group (1, 4 or 12 weeks post i.p. treatment with FgESPs) and each row represents DEmiRNA at 1, 4 or 12 weeks post i.p. treatment with FgESPs. (PDF) [file pntd.0008951.s001.pdf]
