# Supplementary material for: Dysregulation of hepatic microRNA expression in C57BL/6 mice affected by excretory-secretory products of Fasciola gigantica
Source: PLoS Negl Trop Dis. 2020 Dec 17;14(12):e0008951. doi: 10.1371/journal.pntd.0008951 (PMC7775122; doi:10.1371/journal.pntd.0008951)
Supplement: S1 Table — (DOCX) [file pntd.0008951.s002.docx]

**S1 Table. The quality features of small RNA sequencing data.**

| Sample | Reads | Bases | Error rate | Q20 | Q30 | GC content |
| --- | --- | --- | --- | --- | --- | --- |
| E1W_1 | 16316203 | 0.816G | 0.01% | 97.72% | 94.83% | 48.57% |
| E1W_2 | 12023605 | 0.601G | 0.01% | 97.88% | 95.22% | 48.83% |
| E1W_3 | 11369427 | 0.568G | 0.01% | 98.31% | 96.38% | 48.57% |
| C1W_1 | 12046744 | 0.602G | 0.01% | 97.74% | 94.89% | 48.52% |
| C1W_2 | 12979888 | 0.649G | 0.01% | 97.66% | 94.73% | 48.93% |
| C1W_3 | 10327504 | 0.516G | 0.01% | 97.64% | 94.68% | 48.79% |
| E4W_1 | 11064779 | 0.553G | 0.01% | 98.31% | 96.34% | 49.05% |
| E4W_2 | 10766700 | 0.538G | 0.01% | 98.29% | 96.29% | 49.01% |
| E4W_3 | 11258260 | 0.563G | 0.01% | 98.30% | 96.32% | 48.95% |
| C4W_1 | 11944726 | 0.597G | 0.01% | 98.39% | 96.50% | 48.58% |
| C4W_2 | 12185081 | 0.609G | 0.01% | 98.40% | 96.54% | 48.78% |
| C4W_3 | 14597727 | 0.730G | 0.01% | 98.31% | 96.35% | 48.98% |
| E12W_1 | 15888673 | 0.794G | 0.01% | 97.13% | 94.04% | 48.98% |
| E12W_2 | 15153509 | 0.758G | 0.01% | 97.23% | 94.22% | 49.10% |
| E12W_3 | 14139589 | 0.707G | 0.01% | 97.52% | 94.84% | 48.59% |
| C12W_1 | 13774079 | 0.689G | 0.01% | 97.20% | 94.14% | 49.24% |
| C12W_2 | 13413411 | 0.671G | 0.01% | 97.24% | 94.18% | 49.55% |
| C12W_3 | 13649017 | 0.682G | 0.01% | 97.48% | 94.69% | 49.97% |
