# Supplementary material for: Dysregulation of hepatic microRNA expression in C57BL/6 mice affected by excretory-secretory products of Fasciola gigantica
Source: PLoS Negl Trop Dis. 2020 Dec 17;14(12):e0008951. doi: 10.1371/journal.pntd.0008951 (PMC7775122; doi:10.1371/journal.pntd.0008951)
Supplement: S2 Table — (DOCX) [file pntd.0008951.s003.docx]

**S2 Table. Sequence alignment to the reference mouse genome.**

| Sample | Total sRNA | Mapped sRNA | "+" Mapped sRNA | "-" Mapped sRNA |
| --- | --- | --- | --- | --- |
| C1W_1 | 11232437 (100.00%) | 9878167 (87.94%) | 6026364 (53.65%) | 3851803 (34.29%) |
| C1W_2 | 11564296 (100.00%) | 10141280 (87.69%) | 6396167 (55.31%) | 3745113 (32.39%) |
| C1W_3 | 9566664 (100.00%) | 8346326 (87.24%) | 5649204 (59.05%) | 2697122 (28.19%) |
| C4W_1 | 10902647 (100.00%) | 9887638 (90.69%) | 5690765 (52.20%) | 4196873 (38.49%) |
| C4W_2 | 11283648 (100.00%) | 10306665 (91.34%) | 5957164 (52.79%) | 4349501 (38.55%) |
| C4W_3 | 12579130 (100.00%) | 11398388 (90.61%) | 7129160 (56.67%) | 4269228 (33.94%) |
| C12W_1 | 11948874 (100.00%) | 10613993 (88.83%) | 6606491 (55.29%) | 4007502 (33.54%) |
| C12W_2 | 11888202 (100.00%) | 10561431 (88.84%) | 6477497 (54.49%) | 4083934 (34.35%) |
| C12W_3 | 11230026 (100.00%) | 9884624 (88.02%) | 6204084 (55.25%) | 3680540 (32.77%) |
| E1W_1 | 15258454 (100.00%) | 13338318 (87.42%) | 9016218 (59.09%) | 4322100 (28.33%) |
| E1W_2 | 11154500 (100.00%) | 9788823 (87.76%) | 6795077 (60.92%) | 2993746 (26.84%) |
| E1W_3 | 10695028 (100.00%) | 9721564 (90.90%) | 6135335 (57.37%) | 3586229 (33.53%) |
| E4W_1 | 9707760 (100.00%) | 8902026 (91.70%) | 6088984 (62.72%) | 2813042 (28.98%) |
| E4W_2 | 9552110 (100.00%) | 8733893 (91.43%) | 6064990 (63.49%) | 2668903 (27.94%) |
| E4W_3 | 10162833 (100.00%) | 9327671 (91.78%) | 6332785 (62.31%) | 2994886 (29.47%) |
| E12W_1 | 14286236 (100.00%) | 12511344 (87.58%) | 7909071 (55.36%) | 4602273 (32.21%) |
| E12W_2 | 12907407 (100.00%) | 11442425 (88.65%) | 7328943 (56.78%) | 4113482 (31.87%) |
| E12W_3 | 12935306 (100.00%) | 11667309 (90.20%) | 7135171 (55.16%) | 4532138 (35.04%) |

Annotation: "+" represents sense strand of genome; "-" represents antisense strand of genome. C represents control group, E represents ESPs treatment group, and W represents week post injection (wpi). For example, C1W_3 represents sample #3 of 1 wpi control group.
