# Supplementary material for: Dysregulation of hepatic microRNA expression in C57BL/6 mice affected by excretory-secretory products of Fasciola gigantica
Source: PLoS Negl Trop Dis. 2020 Dec 17;14(12):e0008951. doi: 10.1371/journal.pntd.0008951 (PMC7775122; doi:10.1371/journal.pntd.0008951)
Supplement: S3 Table — (DOCX) [file pntd.0008951.s004.docx]

**S3 Table. The differentially expressed miRNAs in the liver tissues of C57BL/6 mice treated with FgESPs.**

| **Groups** | **miRNAs** | **ESP group mean read-count** | **Control group mean read-count** | **log2 Fold Change** | **pval** |
| --- | --- | --- | --- | --- | --- |
| **1 wpe** |  |  |  |  |  |
|  | mmu-let-7a-1-3p | 446.0684961 | 371.600509 | 0.25854 | 0.04475 |
|  | mmu-let-7a-5p | 42328.71027 | 34321.12559 | 0.29593 | 0.021161 |
|  | mmu-let-7d-3p | 1403.911477 | 1050.799873 | 0.40704 | 0.0043424 |
|  | mmu-let-7i-3p | 36.95754317 | 53.12316048 | -0.46693 | 0.043677 |
|  | mmu-miR-101a-3p | 52718.18741 | 75961.35499 | -0.51803 | 4.79E-06 |
|  | mmu-miR-101b-3p | 148612.1307 | 201857.7677 | -0.43526 | 3.82E-05 |
|  | mmu-miR-106b-5p | 822.6612016 | 1228.931323 | -0.54885 | 0.005439 |
|  | mmu-miR-10a-3p | 399.3882826 | 317.8939754 | 0.3265 | 0.0076721 |
|  | mmu-miR-1198-5p | 234.630889 | 176.9903405 | 0.39538 | 0.014248 |
|  | mmu-miR-122-3p | 3882.290475 | 2947.206165 | 0.3879 | 0.0044225 |
|  | mmu-miR-126a-3p | 123397.0443 | 98607.49844 | 0.31345 | 0.03968 |
|  | mmu-miR-1291 | 49.47427879 | 14.82936143 | 1.5643 | 3.10E-08 |
|  | mmu-miR-129-5p | 41.34160978 | 58.11792254 | -0.4711 | 0.037435 |
|  | mmu-miR-129b-3p | 40.15455059 | 57.34676523 | -0.48969 | 0.035431 |
|  | mmu-miR-130a-5p | 35.94077329 | 60.7284376 | -0.71413 | 0.00059127 |
|  | mmu-miR-132-3p | 92.78878552 | 57.02732224 | 0.6665 | 0.0019296 |
|  | mmu-miR-132-5p | 59.45177869 | 88.307605 | -0.53956 | 0.0083712 |
|  | mmu-miR-144-3p | 241.9578871 | 465.6269392 | -0.81565 | 0.0069968 |
|  | mmu-miR-146a-3p | 5.981035874 | 13.95646681 | -0.83405 | 0.035371 |
|  | mmu-miR-148a-5p | 1401.788981 | 1848.255118 | -0.39402 | 1.64E-05 |
|  | mmu-miR-150-5p | 873.9951909 | 632.9166779 | 0.44115 | 0.025786 |
|  | mmu-miR-155-5p | 276.2275344 | 113.1941759 | 1.2143 | 4.06E-09 |
|  | mmu-miR-16-2-3p | 31.8224106 | 51.52537726 | -0.61485 | 0.028717 |
|  | mmu-miR-181a-5p | 2988.608662 | 2395.294851 | 0.31548 | 0.0023473 |
|  | mmu-miR-200c-3p | 186.5265822 | 120.8061182 | 0.60618 | 0.00048723 |
|  | mmu-miR-203-5p | 196.8108843 | 300.0846467 | -0.5845 | 0.0014133 |
|  | mmu-miR-215-5p | 122.6625506 | 447.3728009 | -1.4802 | 2.91E-05 |
|  | mmu-miR-21a-5p | 663522.9518 | 1008002.842 | -0.55217 | 0.023684 |
|  | mmu-miR-23a-3p | 6463.334851 | 4440.18937 | 0.53853 | 2.44E-14 |
|  | mmu-miR-23b-3p | 5892.055204 | 4258.672554 | 0.46648 | 8.81E-16 |
|  | mmu-miR-24-2-5p | 863.7750074 | 1134.971031 | -0.38285 | 0.0062876 |
|  | mmu-miR-26a-5p | 142315.0166 | 109878.5665 | 0.36709 | 0.00096061 |
|  | mmu-miR-26b-5p | 31152.26332 | 22012.02131 | 0.49454 | 7.17E-07 |
|  | mmu-miR-27a-3p | 5912.277963 | 4057.413789 | 0.51745 | 0.0054123 |
|  | mmu-miR-27a-5p | 70.02732027 | 91.86561876 | -0.3768 | 0.028822 |
|  | mmu-miR-27b-3p | 100367.6444 | 84948.06688 | 0.23812 | 0.0077189 |
|  | mmu-miR-293-3p | 0 | 1.783241996 | -0.78252 | 0.034576 |
|  | mmu-miR-29a-5p | 56.91482245 | 97.48087064 | -0.69452 | 0.005129 |
|  | mmu-miR-3074-2-3p | 843.6315874 | 1111.115553 | -0.38604 | 0.0060716 |
|  | mmu-miR-30c-5p | 47632.67168 | 37457.51739 | 0.33776 | 0.015289 |
|  | mmu-miR-322-5p | 428.4071017 | 302.1310892 | 0.46718 | 0.036939 |
|  | mmu-miR-326-3p | 116.1181154 | 171.2238183 | -0.54569 | 0.00095144 |
|  | mmu-miR-331-3p | 63.8838178 | 87.61453508 | -0.44072 | 0.029115 |
|  | mmu-miR-33-5p | 33.34640225 | 68.61673607 | -0.9246 | 0.00095056 |
|  | mmu-miR-340-3p | 187.3507553 | 130.6379639 | 0.50204 | 0.0070523 |
|  | mmu-miR-340-5p | 12225.76951 | 20053.78303 | -0.69941 | 1.66E-08 |
|  | mmu-miR-3470b | 15.27257783 | 31.9051566 | -0.9142 | 0.0021019 |
|  | mmu-miR-34a-5p | 573.8726934 | 1100.663639 | -0.84592 | 0.0012834 |
|  | mmu-miR-3535 | 336.0802927 | 206.7369153 | 0.64224 | 0.010051 |
|  | mmu-miR-361-5p | 373.5104228 | 282.0391623 | 0.39002 | 0.012638 |
|  | mmu-miR-362-3p | 452.6852848 | 361.337636 | 0.32084 | 0.0019546 |
|  | mmu-miR-378a-3p | 40966.41256 | 31943.7561 | 0.34486 | 0.042615 |
|  | mmu-miR-450a-5p | 611.8461466 | 761.4408215 | -0.3106 | 0.016158 |
|  | mmu-miR-490-3p | 5.577581839 | 1.545496493 | 0.9311 | 0.033413 |
|  | mmu-miR-497a-5p | 320.6355002 | 483.1435396 | -0.57425 | 5.33E-06 |
|  | mmu-miR-499-5p | 188.8885816 | 27.4174028 | 1.1789 | 0.0068455 |
|  | mmu-miR-511-3p | 257.7551578 | 349.0035126 | -0.4182 | 0.015346 |
|  | mmu-miR-5121 | 26.5847006 | 14.97035654 | 0.67214 | 0.032784 |
|  | mmu-miR-532-3p | 99.05787968 | 133.6859968 | -0.40623 | 0.024127 |
|  | mmu-miR-574-5p | 137.1077742 | 173.3020794 | -0.32451 | 0.03333 |
|  | mmu-miR-592-5p | 13.17901939 | 42.93179213 | -1.0908 | 0.0093899 |
|  | mmu-miR-664-3p | 115.7909138 | 79.15610172 | 0.53572 | 0.0032865 |
|  | mmu-miR-676-3p | 208.0031461 | 258.9856297 | -0.31057 | 0.031919 |
|  | mmu-miR-872-3p | 88.98657362 | 57.93130588 | 0.55942 | 0.033882 |
|  | mmu-miR-92a-3p | 11858.73593 | 8744.466966 | 0.41936 | 0.022482 |
|  | novel_203 | 0.248438903 | 2.903427724 | -0.85896 | 0.034671 |
| **4 wpe** |  |  |  |  |  |
|  | novel_724 | 1.551936141 | 6.130428909 | -0.95272 | 0.02948 |
|  | mmu-let-7a-5p | 35107.3007 | 46691.9127 | -0.40687 | 1.41E-05 |
|  | mmu-let-7b-5p | 11356.4682 | 14521.83274 | -0.35147 | 4.40E-05 |
|  | mmu-let-7c-5p | 29831.63113 | 40347.03133 | -0.42966 | 3.60E-05 |
|  | mmu-let-7d-5p | 6983.177429 | 8306.601417 | -0.24871 | 0.0014738 |
|  | mmu-let-7e-5p | 1799.884658 | 2316.856842 | -0.35071 | 0.042732 |
|  | mmu-let-7f-5p | 123016.6141 | 206108.4923 | -0.72471 | 4.59E-07 |
|  | mmu-let-7g-5p | 174226.3177 | 263432.6318 | -0.58902 | 2.80E-09 |
|  | mmu-let-7i-5p | 24621.14679 | 38375.40677 | -0.62588 | 2.23E-06 |
|  | mmu-let-7j | 7.131730202 | 14.4106988 | -0.80289 | 0.028782 |
|  | mmu-miR-101b-3p | 136350.6637 | 110181.3968 | 0.30153 | 0.013721 |
|  | mmu-miR-101c | 104.5456152 | 79.40955175 | 0.37444 | 0.036199 |
|  | mmu-miR-103-3p | 12804.22328 | 10979.30308 | 0.21919 | 0.021923 |
|  | mmu-miR-106a-5p | 11.24520922 | 4.03778069 | 0.97671 | 0.016287 |
|  | mmu-miR-106b-5p | 545.6027685 | 419.5774471 | 0.36797 | 0.0082353 |
|  | mmu-miR-107-3p | 3263.74911 | 2577.164506 | 0.33553 | 0.003007 |
|  | mmu-miR-10a-3p | 295.1224997 | 208.7217899 | 0.48681 | 3.52E-05 |
|  | mmu-miR-10a-5p | 39292.5996 | 50823.76153 | -0.36633 | 0.00033649 |
|  | mmu-miR-122-5p | 1093150.789 | 1371411.452 | -0.32354 | 0.00053506 |
|  | mmu-miR-125a-5p | 1525.354133 | 2106.919795 | -0.44117 | 0.030062 |
|  | mmu-miR-126a-3p | 109845.6811 | 95315.97233 | 0.20211 | 0.042265 |
|  | mmu-miR-126a-5p | 9293.051651 | 5033.563803 | 0.84572 | 3.61E-06 |
|  | mmu-miR-126b-3p | 9292.514731 | 5032.282836 | 0.84599 | 3.57E-06 |
|  | mmu-miR-126b-5p | 976.2908293 | 1701.539395 | -0.77282 | 3.26E-06 |
|  | mmu-miR-128-3p | 309.2980046 | 455.350554 | -0.53642 | 0.00075637 |
|  | mmu-miR-134-5p | 15.29027069 | 7.995399521 | 0.71701 | 0.046393 |
|  | mmu-miR-139-5p | 2286.965034 | 3114.225525 | -0.43431 | 0.001483 |
|  | mmu-miR-142a-3p | 816.2879934 | 1130.452582 | -0.44402 | 0.025341 |
|  | mmu-miR-142a-5p | 3849.99786 | 2460.32557 | 0.63116 | 1.29E-06 |
|  | mmu-miR-142b | 816.2879934 | 1130.452582 | -0.44402 | 0.025341 |
|  | mmu-miR-143-3p | 66553.00829 | 86098.42754 | -0.36759 | 5.63E-05 |
|  | mmu-miR-143-5p | 443.76445 | 343.6977044 | 0.35199 | 0.032189 |
|  | mmu-miR-144-3p | 111.3925703 | 207.5512604 | -0.71897 | 0.042332 |
|  | mmu-miR-145a-3p | 1150.575882 | 1496.594826 | -0.36434 | 0.039823 |
|  | mmu-miR-148a-3p | 763385.0547 | 2081063.567 | -1.3621 | 8.06E-11 |
|  | mmu-miR-148a-5p | 1116.573949 | 1676.200772 | -0.56474 | 0.00054629 |
|  | mmu-miR-148b-3p | 6278.986262 | 9680.53841 | -0.62083 | 1.37E-20 |
|  | mmu-miR-148b-5p | 152.7255161 | 111.0226122 | 0.43807 | 0.0042026 |
|  | mmu-miR-150-5p | 503.3361085 | 363.3581928 | 0.44224 | 0.024237 |
|  | mmu-miR-152-3p | 11326.71426 | 6808.148734 | 0.72283 | 3.32E-11 |
|  | mmu-miR-152-5p | 104.0404257 | 170.4546768 | -0.66026 | 0.0027934 |
|  | mmu-miR-155-5p | 142.6361262 | 82.17483876 | 0.67086 | 0.036066 |
|  | mmu-miR-16-5p | 7736.287201 | 5295.368447 | 0.53719 | 3.76E-06 |
|  | mmu-miR-17-5p | 1335.943349 | 910.7965963 | 0.54262 | 1.56E-06 |
|  | mmu-miR-181a-5p | 1831.886712 | 1497.422879 | 0.28854 | 9.84E-06 |
|  | mmu-miR-1839-3p | 56.94625723 | 40.77471 | 0.44203 | 0.039542 |
|  | mmu-miR-1843a-5p | 2359.667507 | 2957.238093 | -0.32312 | 2.66E-06 |
|  | mmu-miR-1843b-5p | 1186.651292 | 1398.868732 | -0.23395 | 0.0097828 |
|  | mmu-miR-191-3p | 21.63443464 | 9.103002145 | 0.95044 | 0.0098218 |
|  | mmu-miR-191-5p | 9446.387426 | 8160.90648 | 0.20972 | 0.00068676 |
|  | mmu-miR-192-3p | 100.8792487 | 64.9300364 | 0.60363 | 0.00088586 |
|  | mmu-miR-193b-3p | 35.86120724 | 20.20194433 | 0.71611 | 0.0079058 |
|  | mmu-miR-194-5p | 138681.2416 | 100080.8457 | 0.45928 | 0.00077995 |
|  | mmu-miR-1947-5p | 10.44624005 | 20.55519397 | -0.81716 | 0.0131 |
|  | mmu-miR-1948-3p | 8.205214624 | 2.832738557 | 0.98802 | 0.020384 |
|  | mmu-miR-195a-5p | 561.6728106 | 428.2740121 | 0.36936 | 0.048648 |
|  | mmu-miR-1964-3p | 21.27868233 | 53.39090475 | -1.1109 | 0.00052723 |
|  | mmu-miR-199a-3p | 14543.04215 | 11119.66341 | 0.37316 | 0.024438 |
|  | mmu-miR-199a-5p | 6256.639491 | 4774.119276 | 0.38569 | 2.30E-05 |
|  | mmu-miR-199b-5p | 466.3776327 | 207.2274533 | 1.0621 | 3.30E-05 |
|  | mmu-miR-19a-3p | 39.59236923 | 81.02287882 | -0.81461 | 0.023398 |
|  | mmu-miR-1a-3p | 103.2483117 | 223.1231225 | -0.95845 | 0.0015991 |
|  | mmu-miR-1b-5p | 102.9741506 | 223.1231225 | -0.96128 | 0.0015729 |
|  | mmu-miR-200a-5p | 49.68144037 | 83.01591057 | -0.63936 | 0.035928 |
|  | mmu-miR-203-3p | 11490.28798 | 10460.86352 | 0.13459 | 0.029159 |
|  | mmu-miR-203-5p | 167.4068383 | 124.183875 | 0.41572 | 0.037855 |
|  | mmu-miR-20a-5p | 3416.187338 | 2521.275213 | 0.42907 | 0.00043988 |
|  | mmu-miR-20b-5p | 24.07602818 | 8.884030987 | 1.0766 | 0.0048338 |
|  | mmu-miR-215-5p | 116.2256083 | 88.37009592 | 0.37784 | 0.03939 |
|  | mmu-miR-218-5p | 40.0981303 | 78.61699479 | -0.90133 | 2.12E-05 |
|  | mmu-miR-219a-5p | 16.10170522 | 7.793706013 | 0.80628 | 0.029003 |
|  | mmu-miR-21a-3p | 23.28974175 | 11.94226114 | 0.82761 | 0.0094765 |
|  | mmu-miR-21a-5p | 445678.5652 | 398836.48 | 0.15895 | 0.043594 |
|  | mmu-miR-221-5p | 193.9571154 | 266.3288536 | -0.4497 | 0.00093513 |
|  | mmu-miR-223-3p | 170.1264782 | 96.37761766 | 0.74884 | 0.0015738 |
|  | mmu-miR-224-5p | 66.17934458 | 86.03335903 | -0.36391 | 0.034509 |
|  | mmu-miR-22-5p | 568.6220795 | 751.9563498 | -0.38865 | 0.015722 |
|  | mmu-miR-23a-3p | 4895.735323 | 3489.983648 | 0.46634 | 0.010989 |
|  | mmu-miR-23b-3p | 5156.716763 | 3881.151227 | 0.39448 | 0.019785 |
|  | mmu-miR-24-2-5p | 668.9917874 | 1041.507992 | -0.60903 | 0.0010197 |
|  | mmu-miR-24-3p | 7065.963526 | 5624.39279 | 0.32176 | 0.013834 |
|  | mmu-miR-26b-5p | 21733.87837 | 25148.40994 | -0.20985 | 3.46E-05 |
|  | mmu-miR-27a-3p | 5557.647907 | 3957.139274 | 0.48249 | 9.36E-06 |
|  | mmu-miR-27b-3p | 93505.96803 | 68014.70392 | 0.44643 | 0.002348 |
|  | mmu-miR-28a-5p | 543.2971261 | 362.2775906 | 0.56029 | 0.00062607 |
|  | mmu-miR-299a-3p | 21.02697292 | 12.49068385 | 0.65341 | 0.045213 |
|  | mmu-miR-29c-5p | 71.36078883 | 131.7915061 | -0.80795 | 0.00071019 |
|  | mmu-miR-3068-5p | 349.118215 | 219.586767 | 0.65866 | 2.11E-10 |
|  | mmu-miR-3074-2-3p | 653.6901138 | 1023.976473 | -0.61763 | 0.00084007 |
|  | mmu-miR-3074-5p | 7019.875704 | 5527.744814 | 0.3367 | 0.011382 |
|  | mmu-miR-30a-5p | 172706.616 | 219929.3743 | -0.3417 | 0.0063537 |
|  | mmu-miR-30c-2-3p | 1393.465093 | 2033.264679 | -0.53792 | 7.66E-08 |
|  | mmu-miR-30c-5p | 36234.98426 | 31862.04165 | 0.18391 | 0.029943 |
|  | mmu-miR-30d-5p | 50070.65448 | 66449.70767 | -0.3996 | 0.0019072 |
|  | mmu-miR-322-3p | 1038.792605 | 780.6929148 | 0.39742 | 0.0076059 |
|  | mmu-miR-322-5p | 177.0712382 | 136.0303192 | 0.36061 | 0.027625 |
|  | mmu-miR-32-3p | 10.06591114 | 24.01651709 | -0.97633 | 0.0080033 |
|  | mmu-miR-32-5p | 486.0363417 | 808.1091077 | -0.6855 | 0.0016869 |
|  | mmu-miR-326-3p | 80.42021651 | 102.9649165 | -0.34901 | 0.032879 |
|  | mmu-miR-328-3p | 627.8539114 | 1031.380669 | -0.70094 | 7.10E-08 |
|  | mmu-miR-33-3p | 36.47865529 | 18.99038176 | 0.80941 | 0.0033331 |
|  | mmu-miR-335-3p | 418.2502368 | 277.4862179 | 0.5689 | 0.00048068 |
|  | mmu-miR-339-3p | 71.14151746 | 31.23829636 | 1.0808 | 4.45E-06 |
|  | mmu-miR-339-5p | 417.7415759 | 238.9691539 | 0.77547 | 3.94E-07 |
|  | mmu-miR-340-5p | 9502.892825 | 15032.90441 | -0.65299 | 9.34E-11 |
|  | mmu-miR-342-3p | 425.077553 | 286.2999621 | 0.52325 | 0.031869 |
|  | mmu-miR-350-3p | 164.6824931 | 111.8531843 | 0.52865 | 0.0039475 |
|  | mmu-miR-3535 | 187.0177377 | 261.7087561 | -0.45647 | 0.019967 |
|  | mmu-miR-361-3p | 1554.711301 | 2262.602199 | -0.53415 | 5.56E-08 |
|  | mmu-miR-361-5p | 302.5342643 | 215.4336445 | 0.47282 | 9.76E-05 |
|  | mmu-miR-362-3p | 310.8774571 | 136.8954466 | 1.1173 | 1.35E-08 |
|  | mmu-miR-365-3p | 744.1899518 | 909.827655 | -0.28405 | 0.0042128 |
|  | mmu-miR-378a-3p | 30220.60458 | 23223.02431 | 0.37587 | 4.91E-05 |
|  | mmu-miR-378c | 6292.381441 | 3582.68508 | 0.7994 | 3.36E-13 |
|  | mmu-miR-379-5p | 214.207834 | 113.0240017 | 0.87143 | 4.25E-05 |
|  | mmu-miR-381-3p | 56.63540831 | 87.56424056 | -0.55519 | 0.03486 |
|  | mmu-miR-382-5p | 12.28519316 | 4.516798748 | 0.99124 | 0.013673 |
|  | mmu-miR-383-5p | 6.582699697 | 18.81930083 | -1.211 | 0.00055002 |
|  | mmu-miR-409-5p | 22.74354443 | 11.08818498 | 0.85316 | 0.012486 |
|  | mmu-miR-411-5p | 153.8593393 | 92.82205737 | 0.7134 | 4.54E-05 |
|  | mmu-miR-423-3p | 2063.136715 | 2910.150785 | -0.49168 | 1.71E-08 |
|  | mmu-miR-423-5p | 861.5513506 | 1341.048524 | -0.62936 | 1.49E-10 |
|  | mmu-miR-425-3p | 90.9015104 | 56.39302866 | 0.64998 | 0.0022001 |
|  | mmu-miR-450a-5p | 448.2122011 | 547.1445095 | -0.281 | 0.011426 |
|  | mmu-miR-451a | 2726.270685 | 4854.468554 | -0.79243 | 3.04E-05 |
|  | mmu-miR-455-5p | 3222.849151 | 6705.663326 | -0.99347 | 2.76E-06 |
|  | mmu-miR-466i-5p | 14.24065456 | 34.53535304 | -0.98502 | 0.0079947 |
|  | mmu-miR-486a-3p | 862.4081441 | 740.1573557 | 0.22045 | 0.0085653 |
|  | mmu-miR-486a-5p | 864.8873511 | 765.8993028 | 0.17586 | 0.030939 |
|  | mmu-miR-486b-3p | 853.6660102 | 732.5823268 | 0.2205 | 0.008999 |
|  | mmu-miR-532-5p | 2492.535398 | 3362.154507 | -0.42305 | 0.00087248 |
|  | mmu-miR-547-3p | 26.68690525 | 13.2900553 | 0.77947 | 0.023349 |
|  | mmu-miR-592-5p | 6.330990293 | 13.86257014 | -0.82128 | 0.040677 |
|  | mmu-miR-598-3p | 197.2404671 | 258.2728171 | -0.38654 | 0.0035914 |
|  | mmu-miR-669c-5p | 17.18730087 | 35.51855225 | -0.9442 | 0.00064222 |
|  | mmu-miR-669o-5p | 2.457109462 | 6.634270438 | -0.87791 | 0.042996 |
|  | mmu-miR-674-3p | 262.6005962 | 204.7570626 | 0.34578 | 0.0034807 |
|  | mmu-miR-690 | 30.40631017 | 50.26157437 | -0.62922 | 0.028943 |
|  | mmu-miR-744-5p | 548.9612276 | 433.5303117 | 0.32683 | 0.031664 |
|  | mmu-miR-7a-5p | 8250.017837 | 12944.91745 | -0.61168 | 0.0035321 |
|  | mmu-miR-7b-5p | 4.903170148 | 19.57564344 | -1.3104 | 0.0017883 |
|  | mmu-miR-871-3p | 0 | 1.621175982 | -0.70245 | 0.049909 |
|  | mmu-miR-872-3p | 67.87920094 | 38.99943669 | 0.74545 | 0.0017148 |
|  | mmu-miR-872-5p | 1114.560294 | 670.835413 | 0.70661 | 5.42E-06 |
|  | mmu-miR-92a-3p | 7803.381651 | 11148.74023 | -0.50691 | 4.48E-06 |
|  | mmu-miR-93-5p | 2327.02748 | 1803.164568 | 0.36402 | 1.56E-05 |
|  | mmu-miR-9-3p | 1.862113111 | 6.627749998 | -0.87523 | 0.049757 |
|  | mmu-miR-9-5p | 99.21557882 | 163.9123233 | -0.69714 | 0.00030315 |
|  | mmu-miR-96-5p | 138.5796264 | 215.2496323 | -0.58591 | 0.010264 |
|  | mmu-miR-98-5p | 808.0981607 | 1300.339465 | -0.67133 | 2.59E-07 |
|  | mmu-miR-99a-5p | 26131.31848 | 46165.34052 | -0.79915 | 2.72E-08 |
|  | mmu-miR-99b-3p | 113.8588772 | 146.8944141 | -0.35936 | 0.030051 |
|  | novel_126 | 31.97627763 | 77.29270468 | -1.1312 | 2.83E-05 |
| **12 wpe** |  |  |  |  |  |
|  | novel_203 | 0.285918177 | 3.381161264 | -0.97282 | 0.020945 |
|  | novel_724 | 0.822837462 | 9.889957332 | -1.5777 | 0.00040689 |
|  | mmu-let-7f-5p | 165756.4041 | 204421.001 | -0.29712 | 0.031746 |
|  | mmu-let-7i-3p | 28.06782629 | 16.89422074 | 0.63877 | 0.049067 |
|  | mmu-miR-10b-5p | 1594.056617 | 507.0490595 | 1.3418 | 0.0010455 |
|  | mmu-miR-125b-2-3p | 121.1041048 | 93.16284124 | 0.36279 | 0.045505 |
|  | mmu-miR-125b-5p | 4328.815014 | 3755.68041 | 0.20336 | 0.023506 |
|  | mmu-miR-126a-3p | 98776.85043 | 114580.1221 | -0.21264 | 0.014022 |
|  | mmu-miR-1291 | 25.54441718 | 12.7589112 | 0.83947 | 0.025479 |
|  | mmu-miR-136-3p | 61.2750065 | 28.07782059 | 0.90017 | 0.030864 |
|  | mmu-miR-139-5p | 2931.405478 | 3689.533093 | -0.32649 | 0.011776 |
|  | mmu-miR-141-3p | 25.22918156 | 10.15086907 | 1.0477 | 0.013852 |
|  | mmu-miR-148a-3p | 1375811.252 | 1740319.218 | -0.33095 | 0.039104 |
|  | mmu-miR-150-5p | 731.3654196 | 608.4759214 | 0.26183 | 0.026359 |
|  | mmu-miR-155-5p | 213.1231404 | 155.9878303 | 0.43981 | 0.014042 |
|  | mmu-miR-181a-5p | 2024.381969 | 1668.083379 | 0.27631 | 0.0058182 |
|  | mmu-miR-181b-5p | 286.1259841 | 227.6081946 | 0.32709 | 0.006738 |
|  | mmu-miR-182-5p | 965.860211 | 687.8035549 | 0.47076 | 0.021256 |
|  | mmu-miR-184-3p | 54.64031804 | 81.05333198 | -0.52884 | 0.042917 |
|  | mmu-miR-185-5p | 2288.511706 | 3063.828815 | -0.40882 | 0.018279 |
|  | mmu-miR-1981-3p | 49.24893064 | 71.60124182 | -0.51985 | 0.011823 |
|  | mmu-miR-1981-5p | 264.7397156 | 374.4285979 | -0.48905 | 0.00056182 |
|  | mmu-miR-199a-3p | 16753.98268 | 10935.90047 | 0.58961 | 0.0050351 |
|  | mmu-miR-199a-5p | 6649.837683 | 4114.723367 | 0.67566 | 2.74E-05 |
|  | mmu-miR-199b-5p | 419.6424631 | 177.9910603 | 1.1144 | 0.0003423 |
|  | mmu-miR-216b-3p | 5.078932029 | 0 | 1.2814 | 0.01046 |
|  | mmu-miR-216c-5p | 5.078932029 | 0 | 1.2814 | 0.01046 |
|  | mmu-miR-219a-5p | 11.26198378 | 4.12896598 | 1.016 | 0.031446 |
|  | mmu-miR-224-5p | 108.9686932 | 60.1577324 | 0.78329 | 0.0068466 |
|  | mmu-miR-22-5p | 720.6392541 | 885.2502227 | -0.29022 | 0.042429 |
|  | mmu-miR-28a-5p | 348.7764081 | 410.3214968 | -0.23273 | 0.033036 |
|  | mmu-miR-29a-3p | 36776.51044 | 32746.40149 | 0.16655 | 0.02821 |
|  | mmu-miR-29b-3p | 1252.428873 | 963.0653248 | 0.37344 | 0.0059043 |
|  | mmu-miR-300-3p | 27.7233886 | 12.42433869 | 0.94713 | 0.017699 |
|  | mmu-miR-3071-5p | 59.56236828 | 27.74324808 | 0.88108 | 0.034745 |
|  | mmu-miR-30d-3p | 133.1639837 | 164.9594958 | -0.30635 | 0.040074 |
|  | mmu-miR-30e-3p | 1293.25808 | 1569.840373 | -0.27525 | 0.043346 |
|  | mmu-miR-30f | 612.1019741 | 729.1356275 | -0.25009 | 0.033276 |
|  | mmu-miR-324-5p | 27.01879167 | 14.67056868 | 0.79336 | 0.015618 |
|  | mmu-miR-33-5p | 29.18188378 | 14.46069524 | 0.8855 | 0.014295 |
|  | mmu-miR-340-3p | 165.6479074 | 222.9640145 | -0.41441 | 0.013446 |
|  | mmu-miR-345-3p | 115.519476 | 157.3739457 | -0.42588 | 0.046162 |
|  | mmu-miR-362-3p | 197.6892972 | 140.1713309 | 0.47497 | 0.0063541 |
|  | mmu-miR-409-5p | 12.53937712 | 4.303482609 | 1.0863 | 0.023464 |
|  | mmu-miR-451a | 5483.492896 | 7481.269612 | -0.44369 | 1.75E-05 |
|  | mmu-miR-497a-5p | 253.8941267 | 199.0730595 | 0.33959 | 0.041541 |
|  | mmu-miR-5126 | 0.85965799 | 4.775130995 | -1.1118 | 0.034019 |
|  | mmu-miR-542-3p | 297.8103043 | 429.9908884 | -0.50082 | 0.031372 |
|  | mmu-miR-6240 | 0 | 2.70341763 | -1.1628 | 0.017191 |
|  | mmu-miR-7015-3p | 0.852980225 | 4.166406731 | -1.0607 | 0.043192 |
|  | mmu-miR-708-3p | 17.43976428 | 1.090975841 | 1.9836 | 0.00013492 |
|  | mmu-miR-708-5p | 15.93219148 | 0.756403332 | 1.8741 | 0.00035256 |
|  | mmu-miR-96-5p | 208.2114483 | 123.0522821 | 0.7037 | 0.010431 |
|  | mmu-miR-99b-5p | 4725.289823 | 5400.252494 | -0.19114 | 0.034995 |
|  | novel_110 | 76.94940697 | 256.7799345 | -1.354 | 0.0018224 |
